# Micronutrients: Minor yet crucial for symbiotic nitrogen fixation

**DOI:** 10.1016/j.xplc.2025.101345

**Published:** 2025-04-22

**Authors:** Jin-Peng Gao, Chai Hao Chiu

**Affiliations:** 1 Crop Science Centre, Department of Plant Sciences, University of Cambridge, Cambridge CB3 0LE, UK; 2 CAS-JIC Centre of Excellence for Plant and Microbial Science, CAS Center for Excellence in Molecular and Plant Sciences, Chinese Academy of Sciences, Shanghai 200032, China

## Abstract

Nodulation represents a crucial but energy-intensive strategy for legumes to survive in nutrient-poor soils. A recent study by Ren et al. (2025) highlights the significance of micronutrients, particularly iron (Fe), in regulating symbiotic nitrogen fixation, which ensures that nodulation occurs only under favorable environmental conditions.

## Main text

Plants engage in beneficial associations with microorganisms that enhance their ability to efficiently acquire essential nutrients, such as nitrogen (N) and phosphorus (P) (Zhao et al., 2024). While most plant species form arbuscular mycorrhizal (AM) symbiosis, legumes additionally develop root nodule symbiosis with nitrogen-fixing rhizobia. These symbiotic interactions are of profound ecological and economic significance, with the potential to contribute to climate change mitigation and agricultural sustainability.

Extensive research has provided a comprehensive understanding of the symbiotic signaling pathway, the developmental processes of mycorrhization and nodulation, and the N and P regulation of symbiosis (Nishida and Suzaki, 2018; Chiu and Paszkowski, 2019; Li et al., 2022; Jhu and Oldroyd, 2023; Zhao et al., 2024). However, how plants integrate environmental signals and nutrient availability to modulate symbiotic microbial interactions remains poorly understood.

Recent studies highlighted the significance of micronutrients such as iron (Fe) and zinc (Zn) in regulating symbiotic nitrogen fixation and uncovered the underlying molecular mechanism (Lin et al., 2024; Ren et al., 2025). The findings reveal how micronutrients coordinate nodulation with plant development and optimize N delivery in legumes, offering potential strategies for improving crop productivity under nutrient-limited conditions.

## Iron sensor directly regulates nodulation signaling

Iron is a key component of diverse proteins and enzymes, playing critical roles in fundamental processes, including photosynthesis, respiration, and oxidative stress responses. The essential role of Fe homeostasis in root nodules stems from its pivotal role as a co-factor for two crucial proteins in symbiotic nitrogen fixation: nitrogenase and leghemoglobin (Hanikenne et al., 2021; Ito et al., 2024; Zhou et al., 2024).

**Figure 1:**
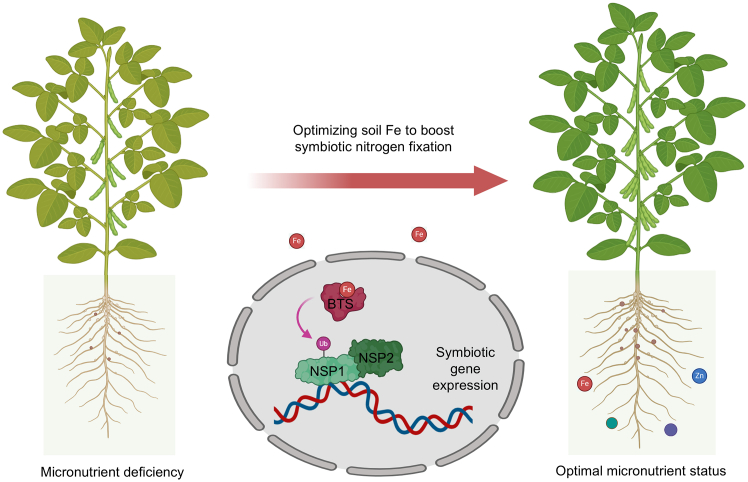
A model illustrating the role of micronutrients, particularly iron, in regulating symbiotic nitrogen fixation in soybean. While multiple micronutrients could be involved in the regulation of nodulation, iron (Fe) serves as a key example during soybean nodulation. Among these micronutrients, Fe availability in the soil is sensed by BTS, which in turn monoubiquitinates NSP1, enhancing symbiotic gene expression. This mechanism was revealed in Ren et al. (2025), and the model was created with BioRender.com.

The recent work by Ren et al. (2025) revealed an interesting regulatory mechanism in which iron directly controls nodulation signaling (Figure 1). In soybean, Fe deficiency (and also toxicity) severely impairs both the development of infection thread and nodule numbers and dramatically suppresses the expression of *NODULE INCEPTION* (*NIN*), a master regulator essential for nodulation. While the expression level of *NODULATION SIGNALING PATHWAY 1* (*NSP1*), a central regulator upstream of *NIN*, remained unchanged, both the protein abundance and transcriptional activity of NSP1 were markedly reduced. To overcome the limitation posed by the complete nodulation deficiency in *nsp1a/b* double mutants, Ren et al. (2025) generated a knockout mutant of *nsp1a* that maintains partial nodulation capacity. The inhibitory effects of Fe limitation on nodulation were largely abolished in the *nsp1a* mutant, indicating that NSP1 plays a role in this regulatory pathway.

Through a yeast two-hybrid screen, Ren et al. (2025) identified BRUTUS (BTS) as an NSP1a-interacting protein. BTS is a well-known iron sensor in many species (Guo et al., 2022). Biochemical and genetic evidence revealed that BTS, functioning as both an iron sensor and E3 ubiquitin ligase, promotes nodulation by monoubiquitinating NSP1a. This post-translational modification enhances NSP1a stability and transcriptional activity and appears to be conserved among legumes. However, the downstream effects of NSP1 monoubiquitination on its interactions with various proteins require further investigation. Particularly, NSP1 and NSP2 can form both heterodimers and homodimers, which are crucial for symbiotic signaling and plant development (Li et al., 2022; Yuan et al., 2023).

Notably, a salt-induced GSK3-like kinase has been shown to phosphorylate NSP1, reducing its DNA-binding activity (He et al., 2021), while an NAC transcription factor interacts with NSP1 and promotes *NIN* expression under salt stress (Wang et al., 2022). Given that soil salinization significantly reduces Fe availability, this raises the intriguing possibility of crosstalk between Fe-dependent regulation mediated by BTS and other stress-responsive pathways to ensure nodulation under favorable conditions. The interplay between Fe homeostasis and salt stress in regulating nodulation under complex environmental conditions requires further investigation.

## Micronutrient signaling bridges nutrient homeostasis and microbial associations

It is well established that iron allocation and signaling are tightly linked to N homeostasis. In soybean nodules, inorganic N disrupts Fe homeostasis through the inhibition of Fe transporters, highlighting the importance of Fe:N balance for efficient symbiotic nitrogen fixation (Zhou et al., 2024). Furthermore, IRON MAN peptides, whose expression is controlled by nitrogen status and NIN, play an essential role in orchestrating nodulation by regulating iron accumulation in root nodules, as well as other nitrogen-associated physiological processes (Ito et al., 2024).

In rice (*Oryza sativa*), the orthologs of BTS interact with and ubiquitinate the PHOSPHATE STARVATION RESPONSE (PHR) transcription factor OsPHR2 (Guo et al., 2022), linking intertwined Fe and P homeostasis and signaling with AM symbiosis (Shi et al., 2021; Das et al., 2022). In addition, the NSP1–NSP2 complex regulates strigolactone biosynthesis, which is essential for P/N starvation responses and mycorrhization (Li et al., 2022; Yuan et al., 2023). These findings reveal the intriguing connection between micro- and macronutrients and raise the possibility that the BTS–PHR2 or BTS–NSP1 interaction is also important in the plant regulation of the AM endosymbiosis.

In addition to Fe, Zn is another essential micronutrient that regulates nitrogen fixation through the Zn-sensing transcriptional regulator FIXATION UNDER NITRATE (FUN) (Lin et al., 2024). Under Zn-sufficient conditions, FUN forms inactive filaments, acting as a molecular storage system. However, when Zn levels decline in response to high N availability, the filaments dissociate, releasing active FUN. Active forms of FUN directly target multiple pathways to trigger nodule breakdown, effectively shutting down nitrogen fixation. This Zn-dependent mechanism fine-tunes nitrogen fixation in response to both internal Zn:N ratios and external N availability, enabling plants to adapt to changing nutrient conditions. While Fe and Zn are distinct trace elements, as reviewed, Fe could interact with Zn and other elements involved in nutrient homeostasis in plants (Hanikenne et al., 2021).

Fe and Zn are not only essential for metabolic processes in plants and microbes but also act as signaling molecules that fine-tune symbiotic efficiency (Lin et al., 2024; Ren et al., 2025). The discovery of BTS (a Fe sensor) and FUN (a Zn sensor) in regulating root nodule symbiosis sets the scene for how plants and microbes utilize micronutrient sensing to optimize nitrogen fixation under varying environmental conditions. Other micronutrients, such as magnesium (Mg) and molybdenum (Mo), which are essential for nodule function (Tejada-Jimenez et al., 2017; Nishida and Suzaki, 2018; Cao et al., 2022), may also participate in these coordination processes. These studies collectively highlight the critical roles of micronutrients not only in mediating the interplay between macronutrient availability and plant growth but also in shaping plant–microbe interactions.

## Concluding remarks

Our knowledge of the genetic mechanisms underlying root nodule symbiosis under variable nutrient environments has significantly advanced. Recent findings discussed here highlight the potential of optimizing soil micronutrient levels, particularly Fe and Zn, to enhance symbiotic nitrogen fixation efficiency in leguminous crops such as peas and beans (Figure 1). Such knowledge can be integrated into fertilization management programs and could offer a promising strategy to enhance crop productivity through monitoring and optimizing nutrient composition. Nevertheless, field trials will be required to demonstrate the true potential of this approach. Finally, a deeper understanding of how plants perceive complex environmental signals and coordinate microbial associations could lead to improved crop stress resilience.

## Funding

This work was supported by the 10.13039/501100001809National Natural Science Foundation of China (32200208 to J.-P.G.) and a grant to the University of Cambridge by the 10.13039/100000865Bill & Melinda Gates Foundation and the UK Foreign, Commonwealth and Development Office (grant no. OPP1172165), known as the Enabling Nutrient Symbioses in Agriculture (ENSA) project. C.H.C. was supported by the 10.13039/501100001352National University of Singapore Overseas Postdoctoral Fellowship (NUS-OPF) and Biotechnology and Biological Sciences (grant no. BB/X011933/1).

## Acknowledgments

We are grateful for the support from Professor Giles Oldroyd. J.-P.G. thanks Xiaoyan Shi for her support. No conflict of interest declared.

## Author contributions

J.-P.G. wrote the manuscript with input from C.H.C. Both authors read and approved the final version of the manuscript.
